# Inhibitory effect of caffeic acid on ADP-induced thrombus formation and platelet activation involves mitogen-activated protein kinases

**DOI:** 10.1038/srep13824

**Published:** 2015-09-08

**Authors:** Yu Lu, Quan Li, Yu-Ying Liu, Kai Sun, Jing-Yu Fan, Chuan-She Wang, Jing-Yan Han

**Affiliations:** 1 Department of gynaecology, Beijing Royal Integrative Medicine Hospital, Beijing, China; 2 Department of Integration of Chinese and Western Medicine, School of Basic Medical Sciences, Peking University, Beijing, China; 3 Tasly Microcirculation Research Center, Peking University Health Science Center, Beijing, China; 4 Key Laboratory of Stasis and Phlegm, State Administration of Traditional Chinese Medicine of the People’s Republic of China.

## Abstract

Caffeic acid (CA), one of the active constituents of *Radix Salvia miltiorrhiza*e, exhibits antioxidant and anti-inflammatory activities. However, few studies have assessed the ability of CA to inhibit platelet mediated thrombus generation *in vivo*. In this study, we investigated the antithrombotic effect of CA in mouse cerebral arterioles and venules using intravital microscopy. The antiplatelet activity of CA in ADP stimulated mouse platelets *in vitro* was also examined in attempt to explore the underlying mechanism. Our results demonstrated that CA (1.25–5 mg/kg) significantly inhibited thrombus formation *in vivo*. *In vitro*, CA (25–100 μM) inhibited ADP-induced platelet aggregation, P-selectin expression, ATP release, Ca^2+^ mobilization, and integrin αIIbβ3 activation. Additionally, CA attenuated p38, ERK, and JNK activation, and enhanced cAMP levels. Taken together, these data provide evidence for the inhibition of CA on platelet-mediated thrombosis *in vivo*, which is, at least partly, mediated by interference in phosphorylation of ERK, p38, and JNK leading to elevation of cAMP and down-regulation of P-selectin expression and αIIbβ3 activation. These results suggest that CA may have potential for the treatment of aberrant platelet activation-related diseases.

In normal hemostasis, platelets prevent hemorrhage after injury and thereby preserve vascular integrity. But, aberrant platelet activation triggered by pathophysiological factors can lead to the development and progression of cardiovascular or cerebrovascular disorders such as hypertension, atherosclerosis, thrombosis and ischemic stroke^1^,^2^,^3^,^4^.

At the injured vascular site, the exposed subendothelial collagen and von Willebrand factor (vWF) initiate platelet activation marked by the secretion of prothrombotic factors like adenosine diphosphate (ADP) and thrombin^5^. Platelet activation is further amplified by ADP and adenosine triphosphate (ATP) released from dense granules and adhesive molecules fibrinogen and P-selectin secreted by α-granules^6^. ADP activates G-protein-coupled receptor signaling mediated by two metabotropic purinergic receptors in platelets. The Gq-coupled P2Y1 receptor is responsible for intracellular calcium mobilization, shape change, and initiation of aggregation; the Gi-coupled P2Y12 receptor inhibits adenylyl cyclase and is responsible for the completion of the aggregation by ADP^7^. In addition, ADP-stimulated Gi-coupled P2Y12 receptor activates mitogen-activated protein kinases (MAPKs) [extracellular signal regulated kinases (ERKs), p38, and c-Jun NH_2_ -terminal kinases (JNKs)] and PI-3k/Akt signaling leading to similar scenario of platelet activation and aggregation^8^.

Currently, the most powerful antiplatelet agents used in the clinic are inhibitors of cyclooxygenase-1 (acetylsalicylic acid or aspirin), the platelet ADP receptor P2Y12 (e.g., clopiodgrel or Plavix), and integrin αIIbβ3 (e.g., abciximab or Reopro)^9^. These agents improve clinical outcomes in large-scale randomized controlled trials. However, the limited efficacy of these drugs in the setting of arterial thrombosis, their adverse effects, cost-to-benefit issues and the drug resistance phenomenon substantiate the need for the development of novel and more efficacious antithrombotic drugs.

Recent studies have revealed the antiplatelet properties of polyphenols isolated from the root of *Salvia miltiorrhiza Bunge*, a herb that is widely used to treat cardiovascular diseases in Asian traditional medicine^10^. For example, salvianolic acid B (SAB) has been shown to inhibit ADP-induced platelet aggregation^11^ and platelet adhesion by interacting with collagen receptor α2β1^12^. 3, 4-dihydroxy-phenyl lactic acid (DLA) and SAB have been reported to suppress the thrombosis induced by photochemical reaction in rat mesenteric venules^13^. Salvianolic acid A (SAA) can inhibit platelet activation and thrombosis via induction of cAMP or inhibition of phosphoinositide 3-kinase^14^,^15^.

Caffeic acid (CA) is one of the major water-soluble components of *Radix Salvia miltiorrhiza.* The chemical structure of CA is illustrated in Fig. 1. Accumulating evidence has confirmed that CA has potent antioxidant and anti-inflammatory activities^16^,^17^. Previous studies showed that CA can inhibit the cyclooxygenase activity and the adhesion protein P-selectin expression in platelets *in vitro*^18^,^19^,^20^. A recent study demonstrated the potential of CA to inhibit collagen-induced platelet aggregation via cAMP-dependent inositol-1,4,5-trisphosphate receptor phosphorylation^21^. However, little is known regarding the role of CA in platelet mediated thrombus generation *in vivo*, and the mechanism thereby the influence of CA on ADP-induced platelet activation has not been fully understood. The aim of this study was to examine the effects of CA on platelet mediated thrombosis in mouse cerebral microvessels and characterize the underlying mechanisms.

## Results

### Influence of CA on mouse cerebral arterial thrombosis

**Figure 2:**
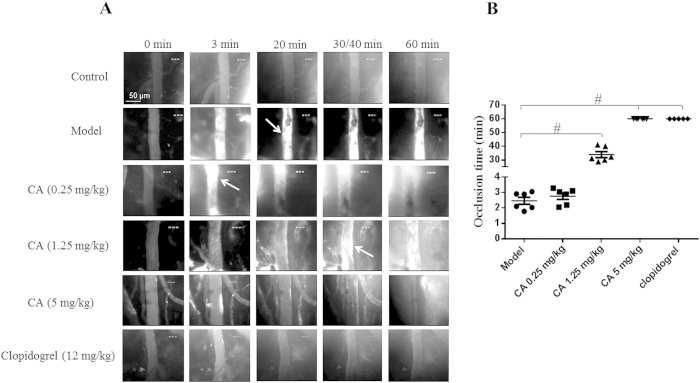
Effects of CA on photochemically induced arterial thrombosis. (**A**) Representative images of thrombus formation in mouse cerebral arterioles induced by photochemical injury in different conditions and at different time points. In order to compare the activities of CA and clopidogrel at a similar blood concentration, 5 mg/kg of CA (MW: 180.16) and 12 mg/kg of clopidogrel (MW: 419.90) were continuously infused through the catheterized femoral vein starting from 20 min prior to the vessel wall injury. This resulted in approximately the same blood concentration of CA and clopidogrel in mice *in vivo*. Arrows indicate irreversible arteriolar vessel occlusion. (**B**) Dot plots of arteriolar occlusion time from five to six experiments. ^#^*p* < 0.05 *vs* Model group.

We first tested the effects of CA on arteriole thrombus formation *in vivo* using intravital microscopy in a mouse cerebral thrombosis model induced by photochemical injury. As shown in Fig. 2A, before photochemical injury, few, if any, fluorescently labeled platelets (bright dots) transiently adhered on the vessel wall. The photochemical injury rapidly provoked platelet adhesion on the arteriolar wall thus building-up of platelet thrombus. The vessel occlusion was often seen in the arterioles within 3 min after injury (Fig. 2B). Pretreatment with CA at 1.25 and 5 mg/kg both significantly decreased platelet deposition and prolonged the time required for thrombus formation and vessel occlusion with 5 mg/kg being more efficient reaching an effect equivalent to clopidogrel (12 mg/kg) (Fig. 2A,B). Treatment with CA at 0.25 mg/kg had no significant effect on the time required for vessel occlusion, though platelet deposition and thrombus formation were attenuated in this case (Fig. 2A,B).

### CA decreases the area ratio of thrombus/venule and inhibits thrombus formation

**Figure 3:**
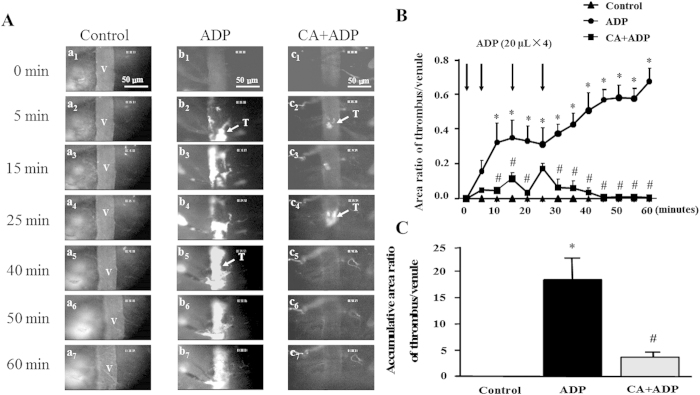
The inhibitory effect of CA on thrombosis in mouse cerebral venule. (**A**) Presented are the representative images of thrombus formation in mouse cerebral venules in control, ADP, and CA + ADP group, at baseline (1), 5 (2), 15 (3), 25 (4), 40 (5), 50 (6), and 60 (7) min, respectively. Selected venules (V) have a diameter of 35–45 μm. Arrows indicate thrombus (T) formed in the venular lumen. (**B**) The effect of CA on time course of the area ratio of thrombus to mouse cerebral venule. (**C**) The effect of CA on accumulative area ratio of thrombus to mouse cerebral venule during 60 min. ADP: the animals were treated with ADP at 0, 5, 15, and 25 min, respectively. CA + ADP: the animals were treated with CA (5 mg/kg) starting from 20 min before thrombus induction. Data are expressed as means ± SEM (n = 10). *p < 0.05 *vs* control group, ^#^*p* < 0.05 *vs* ADP group.

We next assessed the effect of CA on thrombus formation in mouse cerebral venules induced by ADP. As shown in Fig. 3A, only few of fluorescently labeled platelets (bright dots) were detected transiently adhered on the cerebral venular wall in all groups before the topical application of ADP (a1, b1, and c1), which persisted in the control group over the entire observation (a2–a7). In contrast, in ADP group, platelet adhesion was rapidly enhanced on the venular wall in mice, followed by a gradual build-up of platelet thrombus, in which fluorescently labeled platelets were seen as bright dots. The thrombus grew rapidly and obstructed partially the lumen of the vessel in a short time, developing to a comparable size to the venular diameter until it flowed downstream (b2–b4). The blood flow decreased as the thrombus grew, falling quickly over the last minutes and forming a stable thrombus (b5–b7). In the mice pretreated with CA (5 mg/kg), topical application of ADP-induced platelet adhesion and thrombus formation were markedly attenuated, as shown by the delayed thrombus formation and fewer accumulated platelets (c2–c4). After stopping ADP stimulation, thrombus disrupted into non-uniform fragments, which were quickly carried away by the blood flow (c5–c57).

The effect of CA on dynamics of thrombus formation in mouse cerebral venules exposed to ADP is shown in Fig. 3B. No thrombus formation was observed within the period of observation in the control group. Compared to control group, an immediate increase in the area ratio of thrombus/venule was elicited by ADP application in ADP group, reaching statistically significant at 10 minutes and the then remaining a plateau till 25 minutes, at which time point the last application of ADP caused a further time-dependent increase in the area ratio of thrombus/venule. Of notice, pretreatment with CA reduced the area ratio of thrombus/venule significantly nearly at all time points examined after ADP stimulation, as compared to ADP alone group, particularly after stopping ADP stimulation. In addition, the accumulative area ratio of thrombus/venule was statistically analyzed. As shown in Fig. 3C, CA pretreatment significantly decreased the accumulative area ratio when compared to ADP group.

CA Inhibits ADP-induced platelet aggregation. 

**Figure 4:**
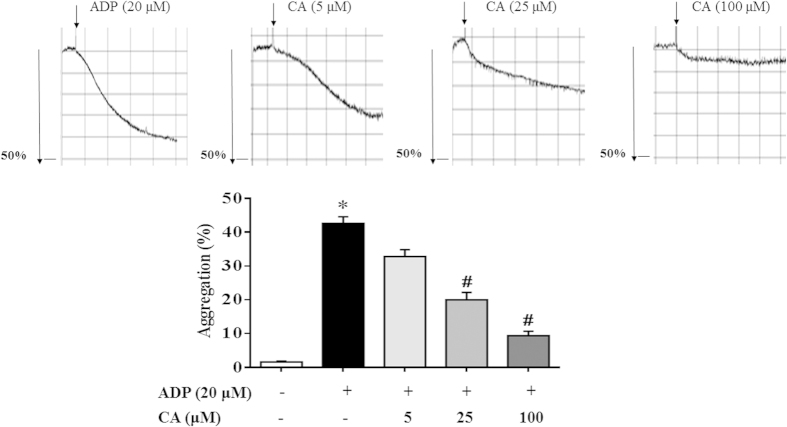
CA inhibits ADP-induced platelet aggregation. Platelets were preincubated with or without CA in the presence of CaCl_2_ (1 mM) at 37 °C for 20 min followed by the addition of ADP (20 μM). The cells were then incubated for 6 min. After incubation with the agonists, platelet aggregation was quantified and expressed as a percentage. Data are expressed as means ± SEM (n = 10). *p < 0.05 *vs* control group, ^#^*p* < 0.05 *vs* ADP group.

ADP is a well-known agonist for platelet aggregation and thrombus formation. To verify the effect of CA on platelet activation, we performed an *in vitro* mouse platelet aggregation test. As shown in Fig. 4, CA (5–100 μM) revealed a dose-dependent inhibition of platelet aggregation caused by ADP (20 μM). Furthermore, argatroban and picotamide were used to exclude the possibility that the effect of CA on ADP-induced platelet aggregation was due to the inhibition of thrombin secretion or thromboxane A2 (TXA_2_) generation. CA inhibited ADP-induced platelet aggregation as well regardless of the presence of argatroban or picotamide, suggesting a thrombin - and TXA_2_-independent effect of CA (Supplemental Figs 1 and 2).

### CA attenuates ADP-induced platelet granule secretion

**Figure 5:**
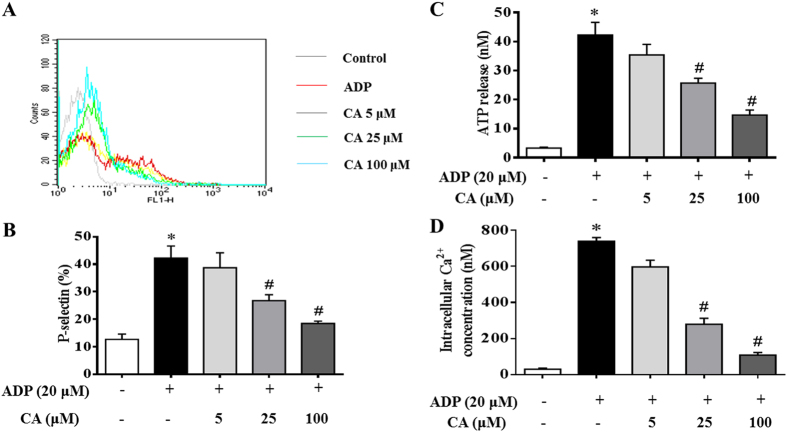
Inhibitory effects of CA on granule secretion, P-selectin expression, ATP release, and Ca^2+^ mobilization in ADP stimulated platelets. Platelets were preincubated with or without CA in the presence of CaCl_2_ (1 mM) at 37 °C for 20 min. The cells were then incubated with ADP (20 μM) for 20 min before P-selectin expression (**A**,**B**), ATP release (**C**), and Ca^2+^ levels (**D**) were analyzed. (**A**) A typical flow cytometry result showing P-selectin expression in different conditions. (**B**) A quantitative assessment of P-selectin expression in different conditions with data from at least four independent experiments. (**C**) A quantitative assessment of ATP release in different conditions. (**D**) A quantitative assessment of intracellular calcium mobilization in different conditions. Data are expressed as means ± SEM (n = 10). *p < 0.05 *vs* control group, ^#^*p* < 0.05 *vs* ADP group.

Since granule secretions are critical marker of platelet activation prior to aggregation, therefore, the effect of CA on ADP-activated granule secretion was examined in the present study. As shown in Fig. 5A,B, stimulation with ADP (20 μM) significantly enhanced P-selectin surface expression in platelets, as compared to that in control untreated platelets. Interestingly, this increase in P-selectin expression was significantly reduced by CA (5–100 μM) in a dose-dependent manner. A similar inhibitory effect of CA was observed for ATP release from platelets (Fig. 5C). Taking together, these results indicate that CA pretreatment inhibits secretory activity of both platelet α-granule and dense granule.

The mobilization of intracellular calcium is widely accepted as a critical step in platelet activation. Therefore, the influence of CA on intracellular calcium mobilization was evaluated. As shown in Fig. 5D, CA prevented the mobilization of intracellular Ca^2+^ induced by ADP, suggesting that the inhibitory effect of CA on platelet activation may be mediated by regulation of cytoplasm calcium level.

### CA inhibits the increase in platelet-derived microparticles (PMPs) induced by ADP

**Figure 6:**
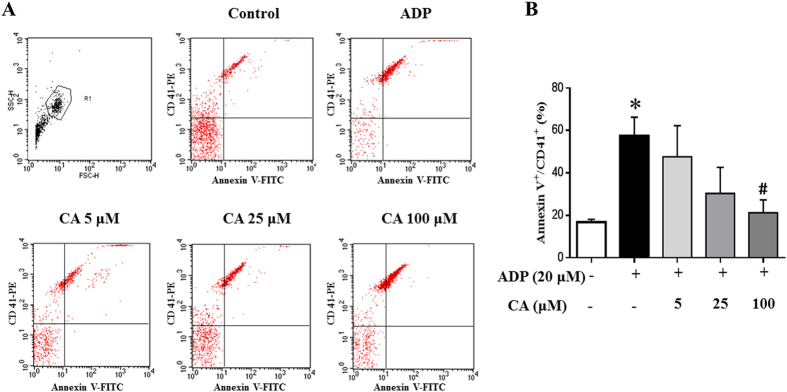
Effect of CA on production of platelet-derived microparticles (PMPs). (**A**) PMPs were detected by flow cytometry with the fluorescence gate designed for Annexin V-FITC and CD41-PE double-positive events. (**B**) Quantitative evaluation of PMPS in different conditions. Data are expressed as means ± SEM (n = 6). *p < 0.05 *vs* control group, #p < 0.05 *vs* ADP group.

In view of the important role of microparticles in regulation of platelet-rich thrombosis, we assessed whether CA could inhibit PMPs production by flow cytometry. The result is presented in Fig. 6, wherein PMPs production is expressed as percentage of Annexin V^+^/CD41^+^. Obviously, ADP stimulation caused a significant increase in PMPs production, which was protected from by pretreatment with CA at 100 μM.

### The effect of CA on cAMP generation in ADP-activated platelets

**Figure 7:**
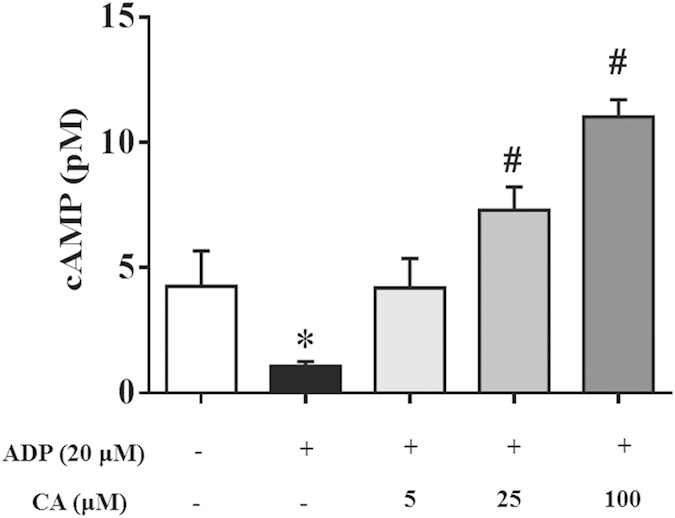
CA elevates cAMP levels in ADP stimulated platelets. Platelets were preincubated with or without CA in the presence of CaCl_2_ (1 mM) at 37 °C for 20 min and then stimulated with ADP for 20 min. cAMP level was determined with a Cyclic AMP XP® Chemiluminescent Assay Kit. CA significantly increased cAMP levels in ADP-stimulated platelets in a concentration-dependent manner. Data are expressed as means ± SEM (n = 10). *p < 0.05 *vs* control group, ^#^*p* < 0.05 *vs* ADP group.

Since cAMP generation and the resulted protein kinase activation are known to inhibit platelet activation, we next determined whether CA alters cAMP levels in ADP-activated platelets. As shown in Fig. 7, the cAMP level in the ADP stimulated platelets was significantly decreased compared with that in the quiescent platelets. Noticeably, CA (5–100 μM) significantly increased the cAMP level in the platelets exposed to ADP in a dose-dependent manner.

### CA inhibits conformational change of integrin αIIbβ3 in ADP-activated platelets

**Figure 8:**
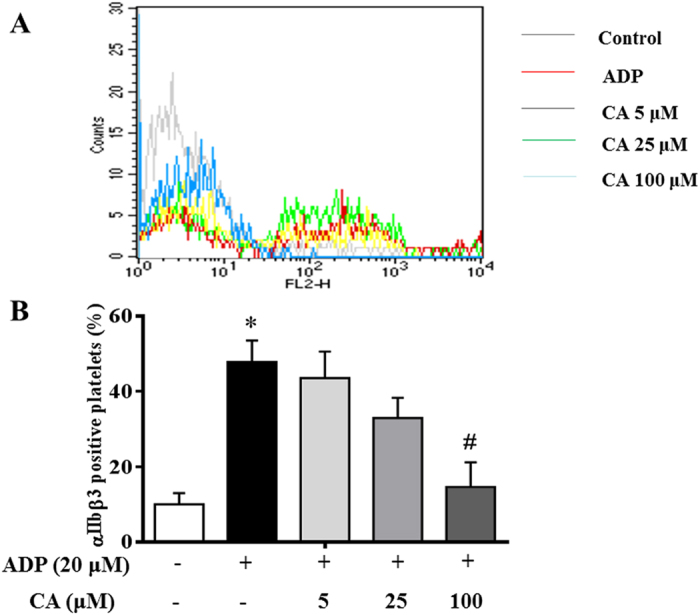
CA inhibits ADP-induced conformational changes in platelet integrin αIIbβ3. Washed platelets were preincubated with CA or vehicle at 37 °C for 20 minutes followed by addition of fluorescent anti-integrin αIIbβ3 monoclonal antibody (JON/A), and stimulated with ADP for 20 min. Positive αIIbβ3 expression was detected by flow cytometry. (**A**) Result from a typical experiment. (**B**) Quantitative evaluation of the data from at least four independent experiments. Data are expressed as means ± SEM. *p < 0.05 *vs* control group; ^#^*p* < 0.05 *vs* ADP group.

The interaction of agonists with platelet receptors triggers inside-out signals that result in conformational changes in the αIIbβ3 integrin, leading to the exposure of neoepitopes and facilitating fibrinogen binding. One of the newly exposed epitopes is recognized by antibody JON/A and the binding of JON/A to the platelet surface is used as a measure of αIIbβ3 integrin activation. Figure 8A shows the fluorescence signal of PE-conjugated JON/A bound to the activated αIIbβ3 receptor of platelets in the presence of various concentration of CA. ADP (20 μM) stimulation evoked a significantly greater αIIbβ3 activation in platelets compared with control group. On the other hand, CA pretreatment markedly suppressed ADP-induced activation of αIIbβ3 being statistically significant at 100 μM (Fig. 8B). This result suggests that CA pretreatment hinders the conformational changes in αIIbβ3 that are required to reveal the binding site for fibrinogen and, most likely, post-ligand occupancy events such as changes in the shape and spreading of the platelets (Supplemental Fig. 3).

### CA attenuates ADP-activated platelet p38, ERK and JNK phosphorylation

**Figure 9:**
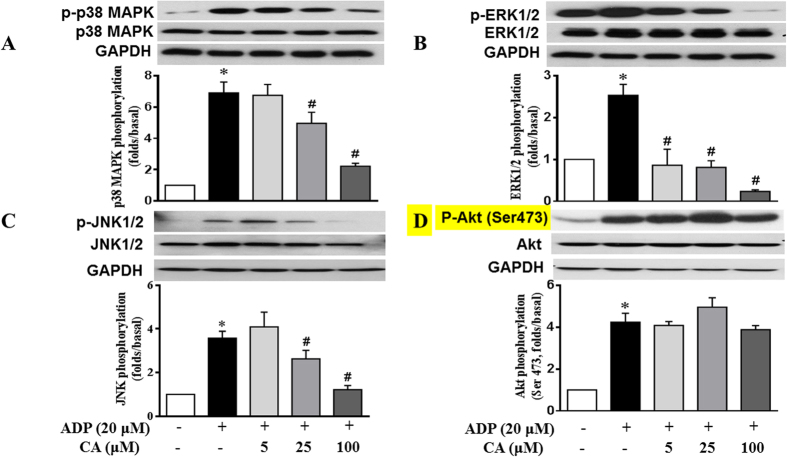
CA attenuates ADP-activated platelet p38MAPK, ERK and JNK phosphorylation. Washed platelets were incubated with CA or vehicle for 20 min and then stimulated with ADP for 20 min. Platelet proteins were then extracted and specific antibodies were used to measure the levels of total and phosphorylated p38 (**A**), ERK (**B**), JNK (**C**), and Akt (**D**). CA attenuated the phosphorylation of p38, ERK and JNK but not of Akt (Ser473) in the activated platelets. Images are representative of three separate experiments. The relative protein expression levels were quantified by Quantity One software. Data represent the mean ± SEM of at least three independent experiments performed in triplicate. *p < 0.05 *vs* control group; ^#^*p* < 0.05 *vs* ADP group.

Phosphorylation of MAPKs, including ERK, p38, and JNK, and Akt in platelets are closely associated with platelet activation and aggregation. Hence, we determined whether CA inhibited MAPKs and Akt phosphorylations in ADP stimulated platelets. The immunoblotting analysis revealed that treatment with ADP (20 μM) provoked marked p38, ERK, JNK and Akt phosphorylation in mouse platelets. Pretreatment with CA dose-dependently suppressed ADP-induced p38 MAPK (Fig. 9A), ERK (Fig. 9B) and JNK (Fig. 9C) phosphorylation. CA did not affect phosphorylation of Akt (Ser473) (Fig. 9D), but significantly reduced phosphorylation of P-Akt (Thr 308) (Supplemental Fig. 4). These data suggest that the MAPKs (ERK, p38, and JNK) and Akt signaling pathways are implicated in the effects of CA.

## Discussion

The present study demonstrated that CA is a potent inhibitor of platelet activation both *in vivo* and *in vitro*. In a well-established mouse model of acute platelet mediated thrombosis, CA significantly reduced the thrombus formation in mouse cerebral arterioles after photochemical stimulation, as well as the area ratio and accumulative area ratio of thrombus to venule and cerebral venular thrombus formation induced by ADP topical application. *In vitro*, CA displayed inhibitory activity on platelet aggregation, calcium mobilization, ATP release, P-selectin expression and integrin αIIbβ3 activation in ADP-activated platelets. Moreover, CA enhanced cAMP production whereas down-regulated ADP stimulated p38MAPK, ERK and JNK phosphorylation.

The most noteworthy finding from the present study was the direct antiplatelet potential of CA *in vivo*. Thromboembolic disorders are still the leading causes of morbidity and mortality in developed societies. Therefore, prophylaxis and treatment of arterial and venous thrombosis are among the main challenges in clinic nowadays. Antiplatelet therapy is the cornerstone in the prevention and treatment of thrombotic diseases. Currently available strategies of antiplatelet therapy are generally based on intervention in the signaling pathway of platelet activation, characterized by mono-agent, mono-target, and mainly irreversible inhibition. These agents each has its own pros and cons with problems such as resistance and drug-drug interaction, etc., and agents with multiple targets are thus proposed to be more superior^22^. Recently, development of antithrombotic agent from medicinal plants with little side effects has attracted much interest. CA, one of the most common phenolic acids frequently occurring in fruits, grains and dietary supplements, is found in traditional Chinese herbs including *Salvia miltiorrhiza*^23^. Previous studies showed that it is a potent antioxidant, able to inhibit ROS production and eliminate active oxygen in macrophages and neutrophils^17^. CA derivatives have been reported to ameliorate oxidative stress in endothelial cells and protect endothelial cells from oxidative stress^24^. A clinic study showed that daily consumption of chicory coffee rich in anti-inflammatory phenols including CA for 1 week substantially decreased the serum macrophage migration inhibitory factor levels of healthy volunteers in parallel with the improved red blood cell deformability, suggesting the encouraging antithrombotic and anti-inflammatory effects of phenolic compounds, including CA^25^. These results, along with the findings of the present study that CA protected thrombus formation at the injury site of mouse cerebral arterioles and in cerebral venules, highly suggest CA as a potential multiple targeting option for antithrombotic therapy. Furthermore, we did not find that ADP or ADP+CA caused any hemorrhage in ADP topically application model, nor hemorrhage induced by CA found in photochemically injured cerebral arteriole model. The assessment of the effect of CA on bleeding time after intravenous administration showed that CA administration (5 mg/kg) did not significantly prolong the tail bleeding time in mice either (Supplemental Fig. 5).

Topical application of ADP is a classic microvascular thrombosis model^26^, in which ADP is typically applied locally to the microvessels of interest by iontophoresis from a micropipette (concentration in the pipette of 10^−2^–10^−3^ M)^27^. This results in platelet adhesion to endothelial cells, which appear normal by transmission electron microscopy^28^. The absence of endothelial denudation in ADP-induced microvascular thrombosis differs from the ferric chloride model, indicating that microvascular thrombosis induced by ADP results from a direct effect on platelet activation^26^. ADP alone is typically used to induce microvascular thrombosis in venules^29^. By applying this well-established mouse model of platelet mediated thrombosis, combined with dynamic microcirculation viewing system, we observed the effects of CA on dynamics of thrombus formation in mouse cerebral cortical venules, and quantitatively analyzed the data via image analysis. Our results showed that CA (5 mg/kg) treatment could significantly reduce the area ratio or accumulative area ratio of thrombus to venule. The thrombus formed in the vessel of CA-treated mice was small and unstable. This suggests that mice treated with CA maintained the ability to form a thrombus, but that thrombus formation was reduced and occurred at a reduced rate. Additionally, our early studies showed that CA could inhibit acute hyperhomocysteinemia-induced endothelia-leucocyte adhesion in mouse cerebral venules^24^ and attenuate rat liver microcirculatory disturbance and oxidative injury through regulation of Sirt3 and mitochondrial respiratory chain^30^. The findings coupled with the present study support the concept that CA modulates thrombotic events through a multitude of pathways.

To provide a foundation for *in vivo* studies in mice and better elucidation of the platelet-inhibiting mechanisms of CA, we examined the effects of CA on ADP-treated mouse platelets *in vitro*. ADP activates two G-protein-coupled purinoceptors, P2Y12 and P2Y1, to transduce its intracellular signaling^31^. P2Y1 receptor is linked to Gq to activate PLCβ with consequent formation of inositol-1,4,5-trisphosphate and Ca^2+^ release on one hand and 1,2-diacylglycerol required for protein kinase C activation on the other. P2Y12 receptor is coupled to Gi and responsible for adenylyl cyclase inhibition^32^. Adenylyl cyclase catalyzes the conversion of ATP to pyrophosphate and cAMP, which is an important mediator for platelet activation signaling. Elevated intracellular cAMP concentrations have been reported to promote platelet stabilization in the presence of agonist-initiated intracellular calcium efflux and platelet aggregation^33^. In this process, cAMP activates a protein kinase that phosphorylates an ATP-dependent calcium-pump leading to Ca^2+^ uptake into the dense tubular system and inhibition of platelet activation cascades^2^,^34^. In the present study, we showed that pretreatment with CA inhibited the Ca^2+^ mobilization while up-regulated the level of cAMP in ADP stimulated platelets, suggesting ADP receptors as the potential targets of CA, albeit this needs further verification.

Upon ADP stimulation, a rapid rise in intracellular Ca^2+^ occurs due to PI3K activation^35^. Increased Ca^2+^ subsequently triggers platelet granule secretion^36^. ATP released from dense granules causes a rapid influx of calcium by sensitizing the ionotropic receptor P2 × 1^37^. In addition, P-selectin released from α granule not only aid leukocyte adhesion to endothelial cells but also is important for inter-platelet aggregation, stabilizing the initial gpIIb/IIIa–fibrinogen interactions, thus allowing the formation of large and stable platelet aggregates^36^,^38^. In line with these data, our results revealed that CA exerted inhibitory effects on calcium mobilization and granule secretion. In addition, CA inhibited binding of single platelets to fibrinogen, an event that is largely dependent on platelet inside-out signaling, and platelet spreading on fibrinogen (Supplemental Fig. 3), which is dependent on outside-in signaling. These results suggested that CA interferes with not only inside-out signaling but also outside-in signaling.

It has been established that MAPKs, ERK2, p38 and JNK1 are present in platelets and activated by various agonists^39^,^40^. Previous studies showed that ERK2, P38 MAPK or JNK1 is phosphorylated and activated by ADP^41^,^42^,^43^. ERK activation following GPIb stimulation leads to integrin αIIbβ3 activation^44^. p38MAPK and ERK are known to have important roles in granule secretion^45^. Furthermore, studies have demonstrated that JNK^−/−^ platelets are associated with decreased integrin αIIbβ3 activation, severe granule secretion impairment and increased bleeding time^39^. Interestingly, the present study observed that CA markedly inhibited ADP-induced activation of p38 MAPK, ERK and JNK, indicating that the modulation of MAPKs signaling pathway may be involved in the anti-platelet activity of CA and that CA suppressed granule secretion, and αIIbβ3 activation through, at least in part, inhibition of the p38MAPK, ERK and JNK pathways. Moreover, Phosphoinositide 3-kinase (PI3K) and Akt are reported to mediate platelet activation via sequential activation of PI3K/Akt, nitric-oxide synthase 3, soluble guanylate cyclase (sGC), and cGMP-dependent protein kinase^46^. A recent repor indicated that PI3Kβ plays an important role in ADP-induced ERK activation^8^. Akt phosphrylation involves Ser473 and Thr308, both contribute to full activation of Akt. In the present study, CA treatment did not affect Akt phosphorylation on Ser 473, but inhibited the phosphorylation on Thr 308 (Supplemental Fig. 4), induced by ADP stimulation, suggesting that antiplatelet activity of CA involves acting on Akt Thr308 thus blocking PI3K/Akt signaling pathway. Nevertheless, the detail mechanism for the antiplatelet activity of CA needs further investigation.

In summary, this study, to the best of our knowledge, is the first to show that CA significantly attenuated thrombus formation *in vivo* in both arteriole and venule. We further demonstrated that CA can directly inhibit *in vitro* platelet activation and aggregation via increased cAMP generation along with the down-regulation of P-selectin expression and αIIbβ3 activation, reduce intracellular Ca^2+^ mobilization, and attenuate phosphorylation of ERK2, p38, and JNK induced by ADP. These findings suggest the potential of CA as a prophylaxis for aberrant thrombosis–related conditions. More studies are required to explore the detailed mechanisms of CA action, evaluate the pharmacokinetic and pharmacodynamic profiles of this herbal component, and optimize the CA dosage before translation to clinic.

## Materials and Methods

### Mice

Male C57BL/6J mice weighing 18 to 22 g, 6–8 weeks of age, purchased from Animal Center of Peking University Health Science Center were housed in temperature- and humidity-controlled rooms. The mice were maintained at a 12-hour light/dark cycle and allowed for free access to rodent chow and tap water. The animal certification number was SCXK (Beijing) 2006–2008. Animal experimental procedures were carried out in compliance with institutional guidelines of the Peking University Animal Research Committee, and experimental protocols were approved by the Committee on the Ethics of Animal Experiments of the Health Science Center of Peking University (Permit Number:16559).

### Reagents

Caffeic acid was purchased from Feng-Shan-Jian Medical Company (purity 98%, Kunming, China). Adenosine diphosphate (ADP), clopidogrel hydrogensulfate acridine red, HEPES and bovine serum albumin (BSA) were all purchased from Sigma (St. Louis. MO, USA). Fura-2/AM was obtained from Invitrogen molecular probes (Eugene, OR, USA). ATP assay kits were purchased from Abcam (Cambridge, MA, USA). Cyclic AMP XP® Chemiluminescent Assay Kit was from Cell Signaling Technology (Danvers, MA, USA). All other reagents were of reagent grade, and deionized water was used throughout the study.

### Antibodies

Fluorescein isothiocyanate (FITC)-conjugated rat anti-mouse CD41 or CD62P (P-selectin) monoclonal antibodies (mAb), Phycoerythrin (PE)-conjugated rat CD 61 mAb and appropriate isotype controls IgG were obtained from BD Biosciences (San Jose, CA, USA). PE-labeled rat anti-mouse Integrin αIIbβ3 mAb (JON/A) was purchased from Emfret Analytics (Wuerzburg, Germany). Polyclonal antibodies against mitogen-activated protein kinase (MAPK) P38, phosphor-p38 (Thr180/Tyr182), ERK, phosphor-ERK (Thr202/Tyr204), JNK, phosphor-JNK (Thr183/Tyr185), Akt, phosphor-Akt (Ser473), and HRP-linked secondary antibodies were all from Cell Signaling Technology (Danvers MA, USA).

### Photochemically induced thrombosis

The animals were anesthetized with urethane (2.0 g/kg body weight, i.p.), and the femoral vein was catheterized for injection of various reagents. The mouse was prostrated with its head fixed between mental strips. The skull was ground down with a hand-held drill at the parietal cortex 4 mm posterior to the bregma and 2 mm lateral to the midline to create an observation window about 0.3 × 0.3 cm which was thin enough to clearly see the arterioles and venules. A single unbranched arteriole (35–45 μm in diameter) was selected under an upright fluorescence microscope (DMLFS; Leica, Mannheim, Germany). Acridine red was injected (5 mg/ml, 12.5 mg/kg) via the femoral vein in order to fluorescently label platelets. After 10 min of basal observation, rose Bengal was injected (20 mg/kg) as a bolus via the femoral vein, and followed by infusion (20 mg/kg/h) for 30 min. Selected arterioles were then exposed to a green light (wavelength 530 nm) for 3 min, resulting in localized and reproducible vessel wall injury. Adhesion and aggregation of fluorescently labeled platelets to the injured site were monitored in real time with a color video camera (TK-C920EC, JVC, Japan). The vessel occlusion was defined as cessation of blood flow that lasted longer than 1 minute due to an occlusive thrombus. Images were transmitted from a super sensitive CCD camera (USS-301, UNIQ Vision, USA) mounted on the microscope onto a color monitor (J2188A, TCL, Huizhou, China), and recorded with a DVD (DVR-R25, Malata, Xiamen, China).

To study the effect of CA on thrombus formation, CA (0.25, 1.25 or 5 mg/kg) or clopidogrel (12 mg/kg) or vehicle was continuously infused through the catheterized femoral vein starting from 20 min prior to the vessel wall injury.

### ADP topical application induced cerebral venular thrombosis

Cerebral venular thrombus was induced by topical application of ADP. For this, the animals were anesthetized with urethane (2.0 g/kg body weight, i.p.), and the femoral vein was catheterized for injection of various reagents. Then, the mouse was prostrated with its head fixed between mental strips. The observation window was prepared as described above with the dura mater uncovered for topical application of ADP. A single unbranched venule (35–45 μm in diameter and 200 μm in length without obvious bend) was selected under an upright fluorescence microscope (DMLFS; Leica, Mannheim, Germany) and exposed to a green light (wavelength 530 nm). The platelets were fluorescently labeled with acridine red (12.5 mg/kg) via the femoral vein catheter. After 10 min of basal observation, 20 μL ADP (20 mM) was added to the observation window at 0, 5, 15, and 25 minutes, respectively. Platelet adhesion and aggregation in the venular lumen was observed in real time with a hypersensitization camera (UNIQ Vision USS-301, Santa Clara, CA USA). The dynamics of the thrombus formation was monitored and recorded under microscope for a period of 60 min. Images were transmitted onto a color monitor (J2188A, TCL, Huizhou, China), and recorded with a DVD (DVR-R25, Malata, Xiamen, China)^47^.

The mice were randomly divided into three groups, 6 animals in each. CA or saline was continuously infused through the catheterized femoral vein starting from 20 min before thrombus induction. In ADP group, animals were infused with saline (6 mL/kg/h) and topically applied with 20 μL ADP (20 mM) at 0, 5, 15 and 25 minutes, respectively. In CA+ADP group, mice were infused with CA (5 mg/kg, 6 mL/kg/h), instead of saline solution, and followed by topical application of 20 μL ADP (20 mM) at the same time points. In control group, the animals were infused with saline (6 mL/kg/h) until the end of the experiment, and topically applied with saline solution, instead of ADP, at the corresponding time points. The observation lasted for 60 min starting from topical application of ADP.

### Evaluation of dynamics of thrombus formation

Based on the recorded images, we examined the thrombus formation in terms of the following parameters:

Area ratio of thrombus/venule, the ratio between the area of thrombus and that of the venule. The thrombus appearance was determined on the basis of the fluorescent-labeled platelets adherent to the venular wall. Area ratio of thrombus/venule was evaluated within 200 μm length of venules and estimated before irradiation and every 5 minutes after irradiation, respectively, using Image-Pro-Plus sofeware 6.0 (Media Cybemetrics Inc, Rockville, MO, USA).

Accumulative area ratio of thrombus/venule, which was defined as the ratio between the accumulative area of thrombus during 60 min of observation and that of the venule.

### Preparation of washed mouse platelets

Mouse blood was drawn from the retro-orbital plexus into a 1.5 mL tube containing 100 μL citrate buffer (25 g sodium citrate, 8 g citric acid, 500 mL H_2_O) and mixed gently. Platelet-rich plasma (PRP) was obtained following centrifugation of blood sample at 500 × g for 5 min. For best recovery of platelets, the complete white phase including some red blood cells was harvested. The PRP samples were again centrifuged at 300 × g for 8 min to remove residual erythrocytes. To obtain washed platelets, acid-citrate-dextrose (ACD, 75 mM sodium citrate, 39 mM citric acid, and 135 mM dextrose, pH 6.5) was added to PRP (1:9), and the suspension of platelets was centrifuged at 1300 ×g for 15 min. The pellet obtained was washed twice with calcium-free Hepes–Tyrode’s buffer (137 mM NaCl, 12 mM NaHCO_3_, 2 mM KCl, 0.34 mM Na_2_HPO_4_, 1 mM MgCl_2_, 5.5 mM glucose, 5 mM HEPES, and 0.35% BSA) and then used as washed platelets. All the procedures for platelet preparations were conducted at room temperature. For flow cytometry studies, platelets from each mouse was analyzed separately without pooling of samples.

### Determination of platelet aggregation

Washed platelets (10^8^/mL) were preincubated for 20 min at 37 °С in the presence of 1 mM exogenous CaCl_2_ with or without various concentrations of CA and then stimulated with ADP (20 μM) for 10 min. The aggregation was monitored using a Chrono-log model 490 optical aggregometer (Havertown, PA, USA) at a constant stirring speed of 1,000 rpm. The aggregation rate was evaluated as an increase in light transmission.

### ATP release assay

Washed platelets were pre-incubated for 20 min at 37 °С with various concentrations of CA and then stimulated with ADP for 20 min. After the reaction was terminated, samples were centrifuged and supernatants were used for ATP assay in a luminometer (Biotek Instruments, Winooski, VT, USA) using an ATP assay kit (Abcam, Cambridge, MA, USA).

### Flow cytometric analysis of platelet activation

Conformational changes of αIIbβ3: Washed platelets were initially treated with CA or vehicle and incubated for 20 min at 37 °С. Flow cytometric analysis was conducted using PE-conjugated JON/A, an antibody directed to the activated conformation of the integrin αIIbβ3 integrin. For this, 25 μL of washed platelets (4 mM10^7^/mL in Hepes–Tyrode’s buffer supplemented with 1 mM CaCl_2_) was mixed with 10 μL of antibody and subsequently stimulated 20 μM ADP for 20 min at room temperature in the dark. The reaction was stopped by addition of 400 μl ice-cold PBS, and samples were analyzed within 30 minutes. For estimation of surface expression levels of total αIIbβ3 integrin, FITC-conjugated anti-CD41 was applied. Samples were analyzed on a FACSCalibur flow cytometer (BD Biosciences), using CellQuest version 3.1f software (BD Biosciences). Both forward and side scatter detectors were set on logarithmic scales, and the analysis gate was set on the major population of platelets. The percentage of positive platelets and their mean fluorescent intensity were calculated from single-parameter histograms of a minimum of ten thousand events from the gated region. The binding of an isotypic control antibody was taken as non-specific binding and was subtracted from the observed geometric mean fluorescence.

P-selectin expression: Platelets were identified by the PE-conjugated anti-CD61, while P-selectin expression was assessed using a FITC-conjugated anti-CD62P. Isotype-matched control antibodies were used to set threshold and exclude nonspecific binding. Washed platelets were pretreated with various concentrations of CA or saline at 37 °С for 20 min prior to addition of ADP (20 μM) or saline for 20 min. All samples were then incubated with antibodies at 37 °С in dark for 20 min. Samples were analyzed immediately on a flow cytometer (FACSCalibur, BD Biosciences). On the FSC vs. SSC plot, a gate was drawn around the platelets and 10,000 platelet events were collected per sample. Platelets were identified as PE-CD61 positive and P-selectin expression was reported as percentage of activated platelets.

### Measurement of intracellular calcium mobilization by Fura 2-AM fluorescence

Washed platelets were incubated with 5 μM of Fura-2/AM for 60 min at 37 °С and washed. The Fura-2-loaded platelets were then pre-incubated with CA for 20 min at 37 °С in the presence of 1mM CaCl_2_, then stimulated with ADP (20 μM) for 20 min. Fluorescence signals were recorded using a fluorescence spectrofluorometer (Biotek Instruments, Winooski, VT, USA). Fluorescence emission was determined at 510 nm, with simultaneous excitation at 340 and 380 nm changing every 1s. The intracellular Ca^2+^ concentrations were calculated using the method developed by Schaeffer^48^.

### Assessment of platelet-derived microparticles (PMPs) production

Platelet-derived microparticles (PMPs) were detected by flow cytometry with modification^49^. Briefly, washed platelets were preincubated for 20 min at 37 °С in the presence of 1 mM exogenous CaCl_2_ with or without CA at various concentrations, and then stimulated with ADP (20 μM) for 20 min. Activated platelets were removed by centrifugation at 1300 × g for 5 min at 20 °C and the supernatants containing PMPs suspensions were collected. All samples were then incubated with CD41-phycoerythrin (PE) and Annexin V-fluorescein isothiocyanate (FITC) antibodies (BD Biosciences, San Jose, CA, USA) in dark for 30 min. Samples were analyzed immediately on a flow cytometer (FACSCalibur, BD Biosciences, San Jose, CA, USA). For PMPs detection, the fluorescence gate was designed for annexin V-FITC and CD41-PE double-positive events. A total of 10,000 platelet events were collected, and the result was expressed as percentage of Annexin V^+^/CD41^+^.

### Determination of cyclic adenosine monophosphoate (cAMP)

Washed platelets were preincubated for 20 min at 37 °С with various concentrations of CA or vehicle in the presence of 1mM CaCl_2_, and then stimulated with ADP (20 μM) for 20 min. The reaction was terminated by addition of 80% ice-cold ethanol. Samples were then centrifuged at 2,000 × g for 10 min at 4 °С and the supernatant cAMP level was determined with a Cyclic AMP XP® Chemiluminescent Assay Kit (Cell Signaling, Danvers, MA, USA).

### Western blot

Aliquots of washed mouse platelets preincubated with various concentrations of CA or saline for 20 min were stimulated by ADP (20 μM) or saline at 37 °С for 20 min. Lysis buffer (20 mM Tris, 150 mM NaCl, 1 mM EDTA, 1 mM EGTA, 1% NP-40, 1% sodium deoxycholate, 2.5 mM sodium pyrophosphate, 1 mM beta-glycerophosphate, 1 mM PMSF, 1 μg/ml leupeptin and 1% protease inhibitor cocktail) was added to obtain the cell lysates which were then centrifuged at 20,000 × g for 30 min. The protein content was determined with a bicinchoninic protein assay kit (BCA; Pierce, Rockford IL, USA). Equal amounts of platelet proteins (30 μg) were resolved in (8 to 12%) SDS-PAGE and transferred to polyvinylidene difluoride (PVDF) membranes in a transfer buffer (25 mM Tris, pH 8.5, 0.2 M glycine, and 20% methanol). Blots were blocked with TBST buffer (150 mM NaCl, 10 mM Tris–HCl, and 0.05% Tween-20) containing 5% nonfat dry milk or BSA and incubated with primary antibodies diluted in blocking solution at 4 °С overnight. The immunoblots were then incubated with horseradish peroxidase (HRP)-conjugated anti-rabbit IgG (1:5,000) and the membranes were visualized using enhanced chemiluminescence detection reagents (Applygen Technologies, Beijing, China). Densitometric analyses of Western blots were performed using the Quantity One image analyzer software (Bio-Rad, Richmond CA, USA). The analysis was performed using volume rectangular tool. An identical area was selected above and below the bands for background subtraction using the global method.

### Statistical analysis

All of the data obtained in this study are shown as mean ± SEM of at least three independent experiments. Statistical significance was determined by one-way analysis of variance followed by Tukey’s t-test using GraphPad Prism software (GraphPad, San Diego, CA, USA). P-values less than 0.05 were considered statistically significant.

## Additional Information

**How to cite this article**: Lu, Y. *et al.* Inhibitory effect of caffeic acid on ADP-induced thrombus formation and platelet activation involves mitogen-activated protein kinases. *Sci. Rep.* **5**, 13824; doi: 10.1038/srep13824 (2015).

## Supplementary Material

Supplementary Information

## Figures and Tables

**Figure 1:**
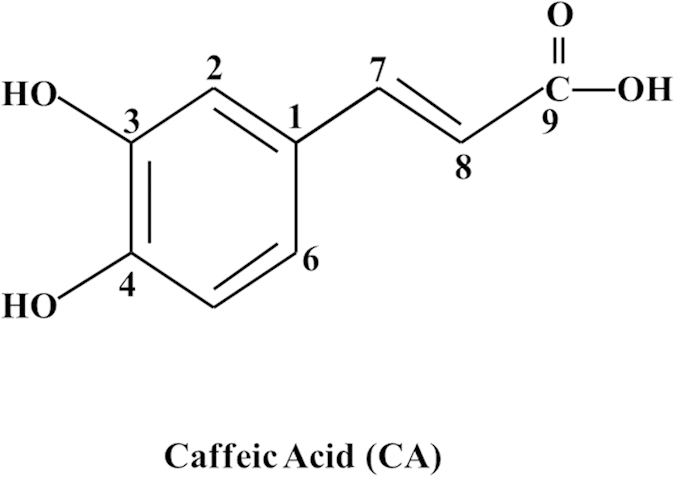
Chemical structure of caffeic acid (CA).
