# Supplementary material for: Inhibitory effect of caffeic acid on ADP-induced thrombus formation and platelet activation involves mitogen-activated protein kinases
Source: Sci Rep. 2015 Sep 8;5:13824. doi: 10.1038/srep13824 (PMC4561902; doi:10.1038/srep13824)
Supplement: Supplementary Information [file srep13824-s1.pdf]

Inhibitory effect of caffeic acid on ADP-induced thrombus formation and platelet activation involves mitogen-activated protein kinases

Yu Lu[1,2,3,#], Quan Li[3,4,#], Yu-Ying Liu[3,4], Kai Sun[3,4], Jing-Yu Fan[3], Chuan-She Wang[2,3,4,*] & Jing-Yan Han[2,3,4,*]

Supplementary material and methods

Platelet aggregation

Washed platelets (10^8/mL) were preincubated for 20 min at 37°C in the presence of 1 mM exogenous $CaCl_2$ with or without CA (100 μM), argatroban (0.005 mg/mL) or picotamide (50 μM), and then stimulated with ADP (20 μM) for 10 min. The aggregation was monitored using a Chrono-log model 490 optical aggregometer (Havertown, PA, USA) at a constant stirring speed of 1,000 rpm. The aggregation rate was evaluated as an increase in light transmission.

Platelet spreading on fibrinogen

Glass coverslips (24 ×50 mm) were coated with 20 ug/mL fibrinogen (Sigma, St Louis, MO, USA) in 0.1 M $NaHCO_3$ (pH 8.3) at 4 ℃ overnight. Washed platelets preincubated with various concentrations of CA at 37 ℃ for 20 min were allowed to spread on the fibrinogen-coated surfaces at 37 ℃ for 90 min. After three washes with PBS, the cells were fixed, permeabilized, and stained with rhodamine-labeled

phalloidine (Life Technologies, Carlsbad, CA, USA). Adherent platelets were viewed with a laser scanning confocal microscope (TCS SP5, Leica, Mannheim, Germany). The spreading area of individual platelet was assessed using Image-Pro-Plus sofeware 6.0 (Media Cybemetrics Inc, Rockville, MO, USA).

Western blot analysis:

Aliquots of washed mouse platelets preincubated with various concentrations of CA or saline for 20 min were stimulated by ADP (20 μM) or saline at 37°C for 20 min. Lysis buffer (20 mM Tris, 150 mM NaCl, 1 mM EDTA, 1 mM EGTA, 1% NP-40, 1% sodium deoxycholate, 2.5 mM sodium pyrophosphate, 1 mM beta-glycerophosphate, 1 mM PMSF, 1 μg/ml leupeptin and 1% protease inhibitor cocktail) was added to obtain the cell lysates which were then centrifuged at 20, 000 × g for 30 min. The protein content was determined with a bicinchoninic protein assay kit (BCA; Pierce, Rockford IL, USA). Western blot analysis was performed routinely, with primary antibodies against Akt, Phospho-Akt (Thr308), and GAPDH (Cell Signaling Technology, Beverly, MA, USA). The bands were detected using ECL detection kit (Applygen Technologies, Beijing, China). Densitometric analyses of Western blots were performed using the Quantity One image analyzer software (Bio-Rad, Richmond CA, USA). The analysis was performed using volume rectangular tool. An identical area was selected above and below the bands for background subtraction using the global method.

Assessment of bleeding time of mouse tail

The tail bleeding model was based on previous methods with minor modifications [1]. CA or saline were administered intravenously 20 min before bleeding. Mice were anesthetized with 20% urethane, and the tail was immersed in 0.9% saline solution at 37 ℃. After 3 min, a distal 2 mm segment of the tail was severed with a razor blade. The tail was immediately re-immersed in warm saline with the tip of the tail 5 cm below the body. The time between the start of transection to bleeding cessation was recorded as the bleeding time. Bleeding cessation was considered to be the time when the flow of blood stopped.

Supplementary results

CA inhibits ADP-induced platelet aggregation independent of thrombin and TXA2

ADP-induced platelet activation and aggregation are potentiated by secondary generated stimuli, including thrombin and TXA_2. To test the role of thrombin and TXA_2 in the effect of CA on ADP-induced platelet aggregation, a platelet aggregation experiment was performed, in which argatroban (thrombin inhibitor) and picotamide (TXA_2 antagonist and TXA_2 synthase inhibitor) were applied. As shown in Supplemental Figure 1 and 2, CA inhibited ADP-induced platelet aggregation significantly irrespective of absence or presence of argatroban and picotamide, implying that the effect of CA on ADP-induced platelet aggregation is independent of thrombin or TXA_2.

Effects of CA on platelet spreading on immobilized fibrinogen

We further determined the effects of CA on platelet spreading on immobilized fibrinogen (Supplemental Figure 3). In the absence of CA, the average surface coverage of spread platelets was 11.76 ± 1.55 μm^2. CA dose-dependently inhibited platelet spreading and reduced the surface coverage.

,

Effects of CA on ADP-induced Akt (Thr 308) phosphorylation

The immunoblotting analysis revealed that treatment with ADP (20 μM) provoked

marked Akt phosphorylation at Thr 308 in mouse platelets. Pretreatment with 100 μM of CA suppressed ADP-induced Akt (Thr 308) phosphorylation (Supplemental Figure 4).

Effect of CA on bleeding time

Since anti-thrombotic activity could be accompanied by the adverse effects such as prolonged bleeding time and impaired blood coagulation, we determined the effect of CA on bleeding time after intravenous administration in mouse. As shown in Supplemental Figure 5, the tail bleeding time of untreated mice was 67 ± 4.2 s. Administration of CA did not significantly prolong the tail bleeding time (78.5 ± 5.2 s).

Figure Legends

Figure S1. Effects of CA on platelet aggregation in the presence or absence of thrombin inhibitor.

Effect of CA on ADP-induced platelet aggregation in the absence or presence of argatroban. Data are expressed as means ± SEM (n=6). $^{\#}p < 0.05$ *vs* argatroban group.

Figure S2. Effects of CA on platelet aggregation in the presence or absence of TXA2 synthase inhibitor.

Effect of CA on ADP-induced platelet aggregation in the absence and presence of picotamide. Data are expressed as means ± SEM (n=6). $^{*}p < 0.05$ *vs* ADP group, $\#p <$ 0.05 *vs* picotamide group.

Figure S3. Effects of CA on platelet spreading on fibrinogen-coated surface.

(A) Representative images from at least three independent experiments with similar results. (B) Mean ± SEM of the average surface area of individual platelets are plotted. $^{*}p < 0.05$ *vs* control group.

Figure S4. Effects of CA on Akt phosphorylation (Thr 308) induced by ADP stimulation.

Western blot mages are representative of three separate experiments. The relative protein expression levels were quantified by Quantity One software. Data represent

the mean ±SEM of at least three independent experiments performed in triplicate. *$p < 0.05$ *vs* control group; $^{\#}p < 0.05$ *vs* ADP group.

Figure S5. Effect of CA on bleeding time of mouse tail

Mice received CA 5 mg/kg (in CA+ Control) or equivalent volume of saline (Control) by intravenous infusion. Tail bleeding time was determined in seconds. Data are expressed as means ±SEM (n=6).

References

1. Greene TK*, et al.* Towards a standardization of the murine tail bleeding model. *J Thromb Haemost* **8**, 2820-2822 (2010).

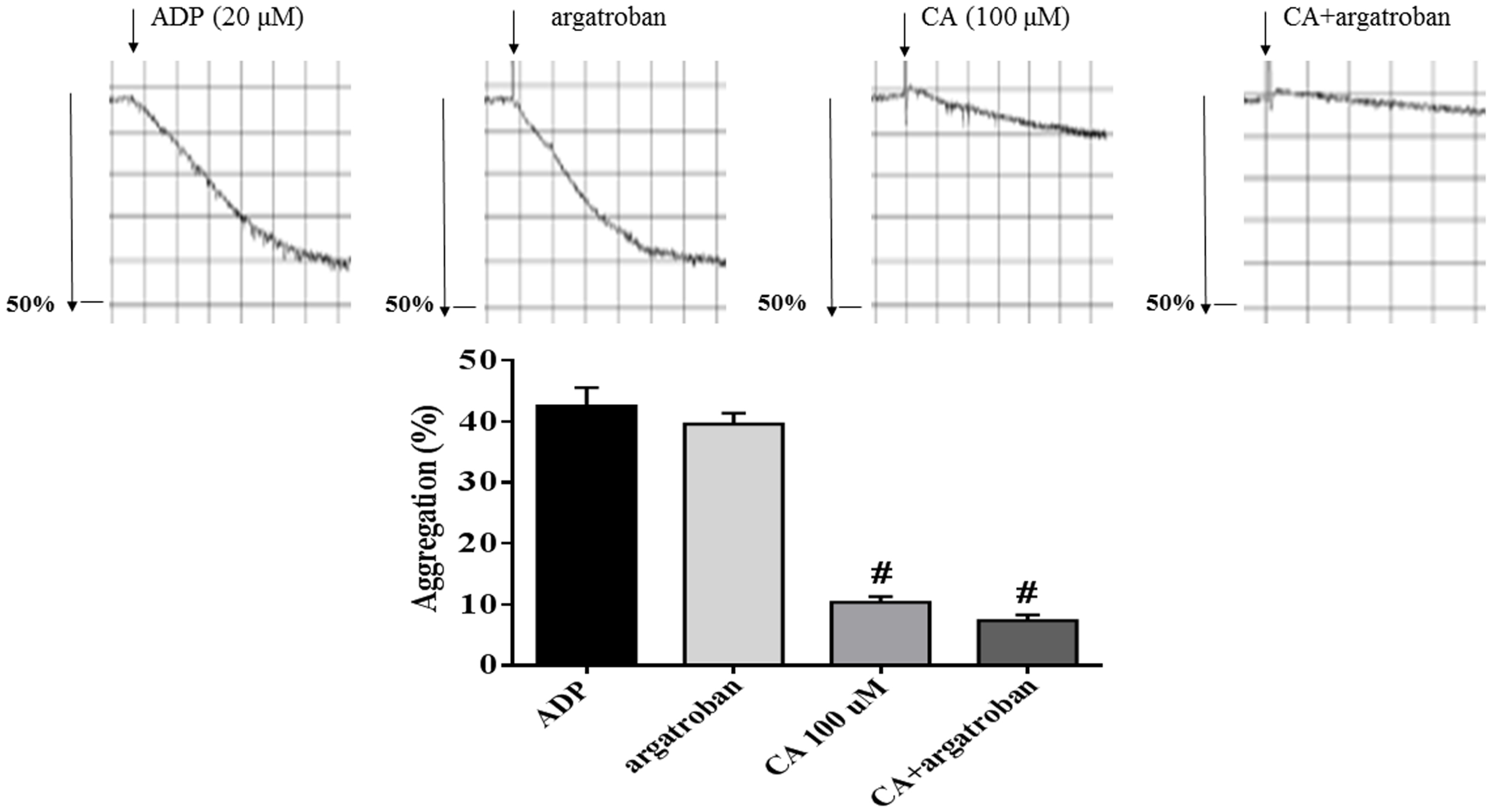

ADP (20 μM)
argatroban
CA (100 μM)
CA+argatroban
50%
50%
50%
50%
Aggregation (%)
50
40
30
20
10
0
#
#
ADP
argatroban
CA 100 uM
CA+argatroban

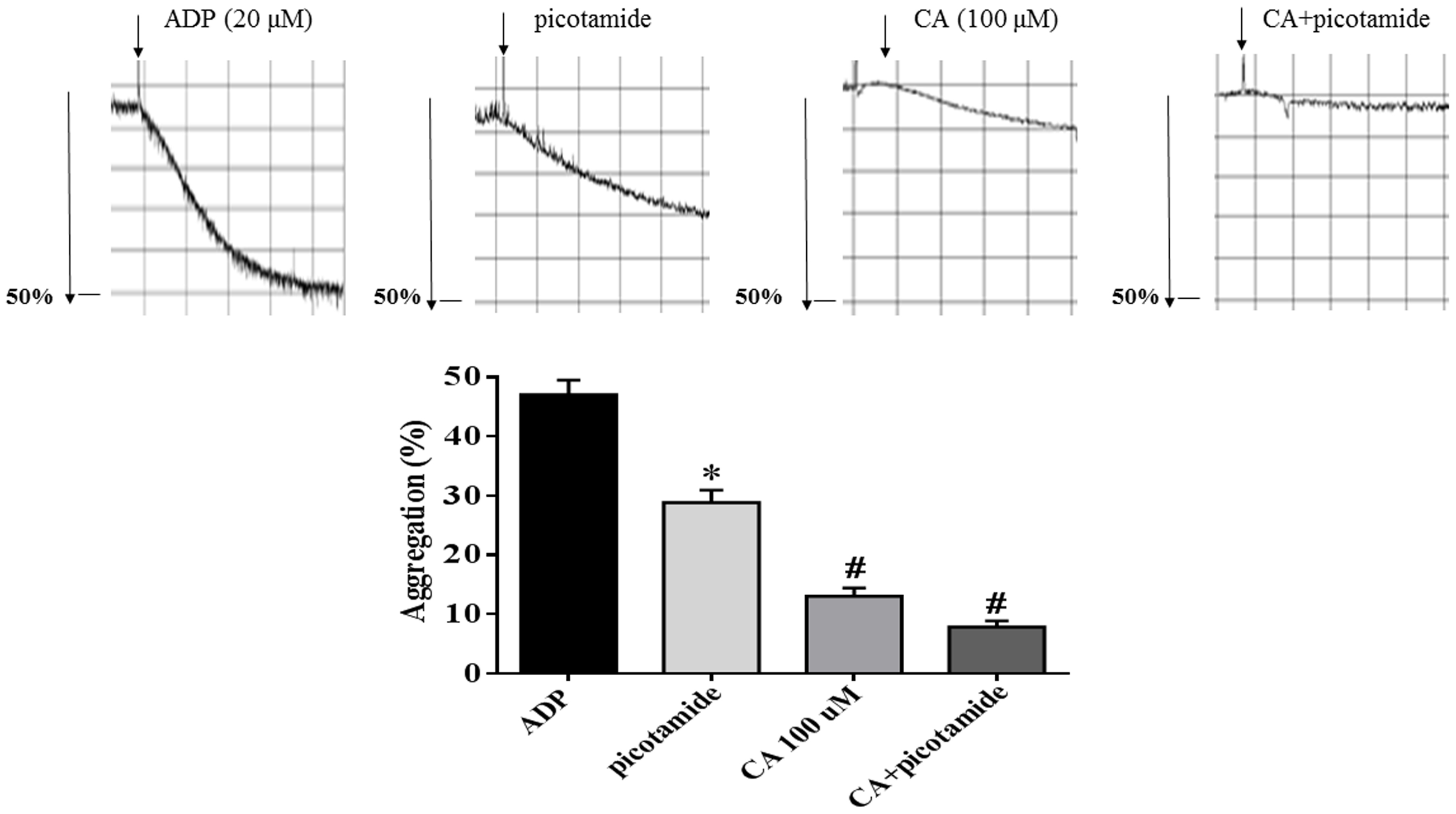

ADP (20 μM)
picotamide
CA (100 μM)
CA+picotamide
50%
50%
50%
50%
Aggregation (%)
50
40
30
20
10
0
*
#
#
ADP
picotamide
CA 100 uM
CA+picotamide

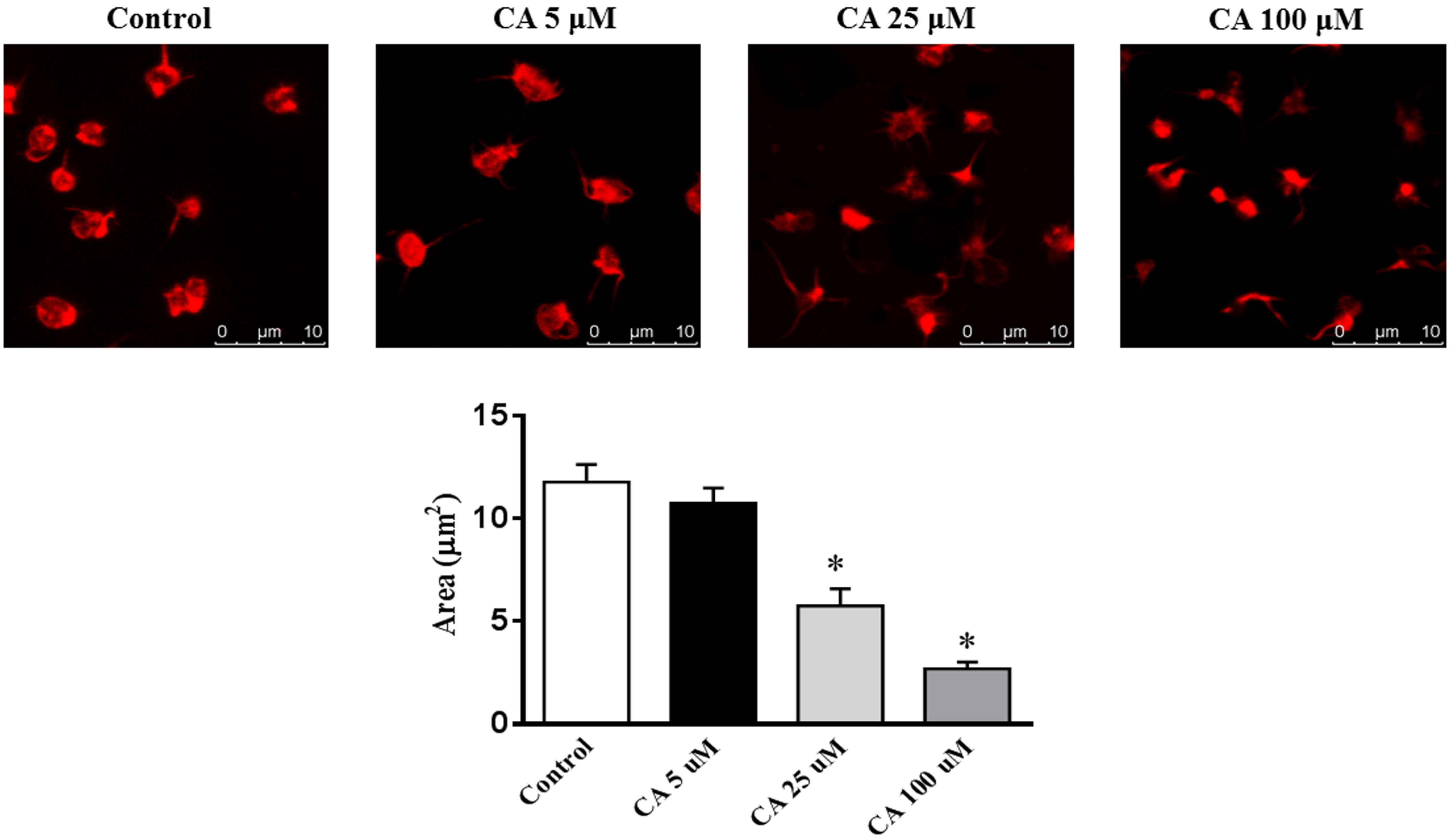
Control
CA 5 μM
CA 25 μM
CA 100 μM
0 μm 10
15
10
5
0
Area (μm²)
*
*
Control
CA 5 uM
CA 25 uM
CA 100 uM

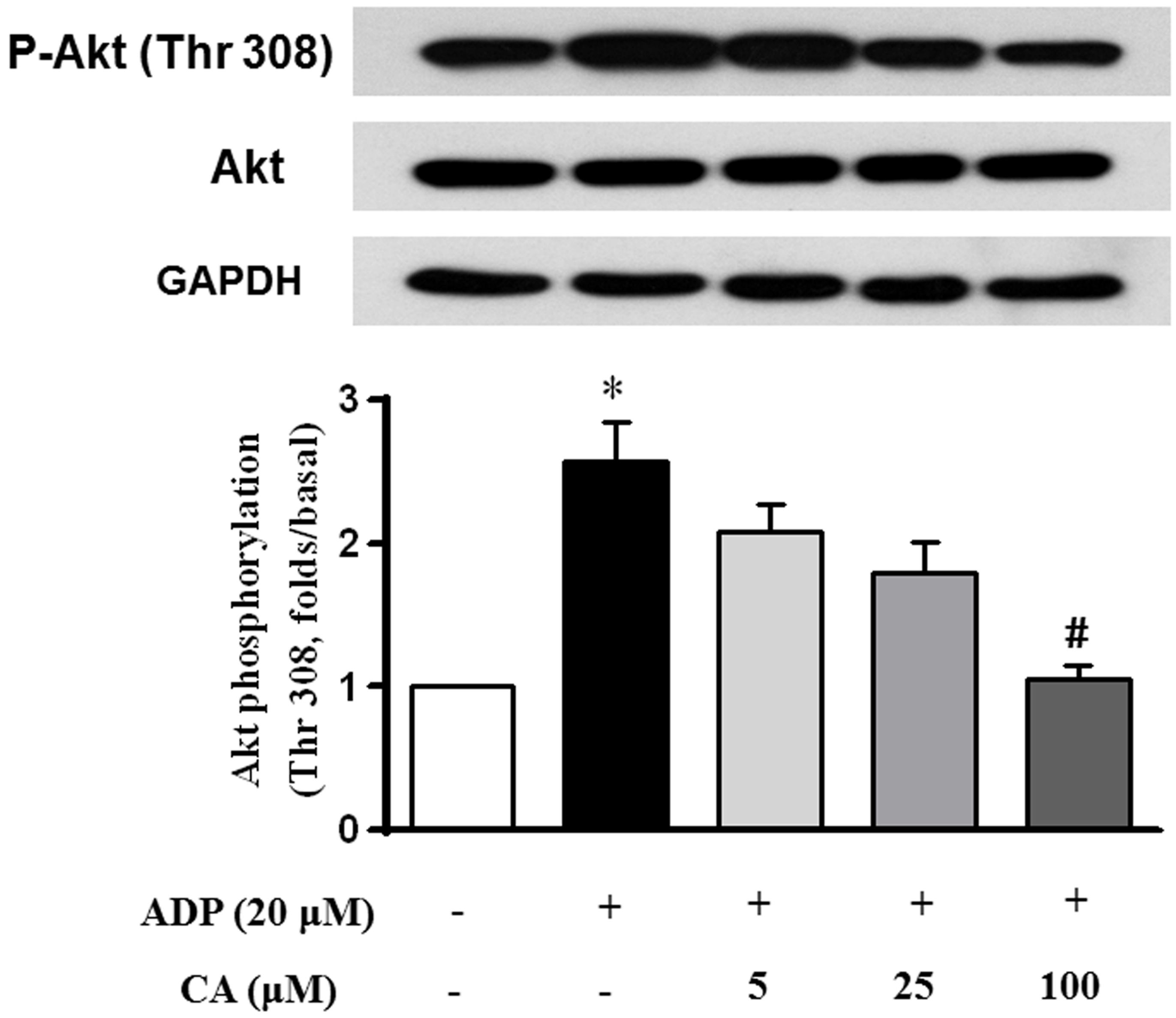
P-Akt (Thr 308)
Akt
GAPDH
Akt phosphorylation (Thr 308, folds/basal)
0
1
2
3
*
#
ADP (20 μM)
- + + + +
CA (μM)
- - 5 25 100

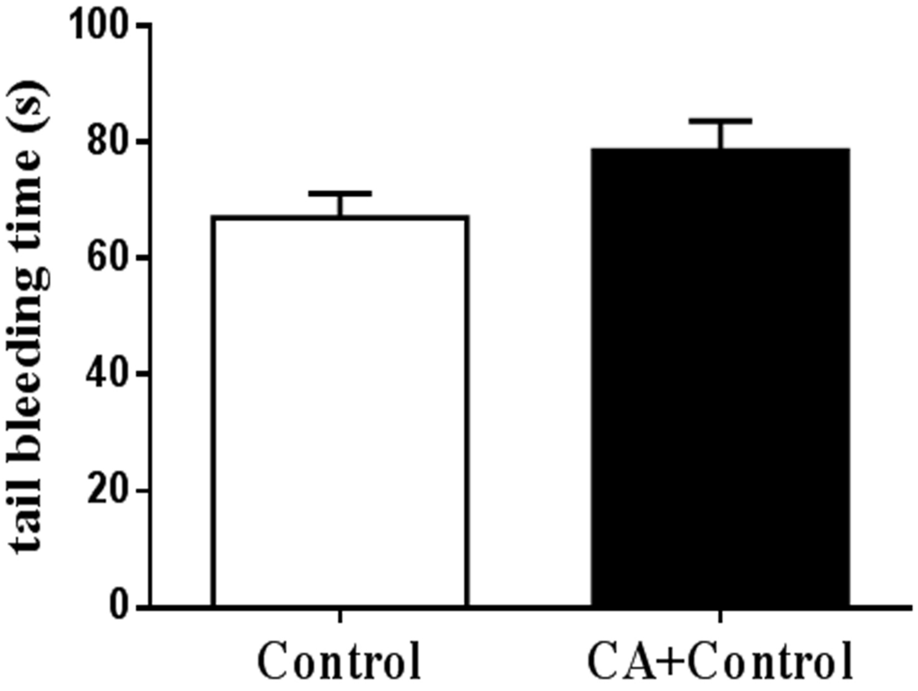

tail bleeding time (s)
100
80
60
40
20
0
Control
CA+Control
